# Supplementary material for: Head-to-Head Comparison of Aptamer- and Antibody-Based Proteomic Platforms in Human Cerebrospinal Fluid Samples from a Real-World Memory Clinic Cohort
Source: Int J Mol Sci. 2024 Dec 31;26(1):286. doi: 10.3390/ijms26010286 (PMC11720409; doi:10.3390/ijms26010286)
Supplement: Supplementary file 1 [file ijms-26-00286-s001.zip › ijms-3312862-supplementary.pdf]

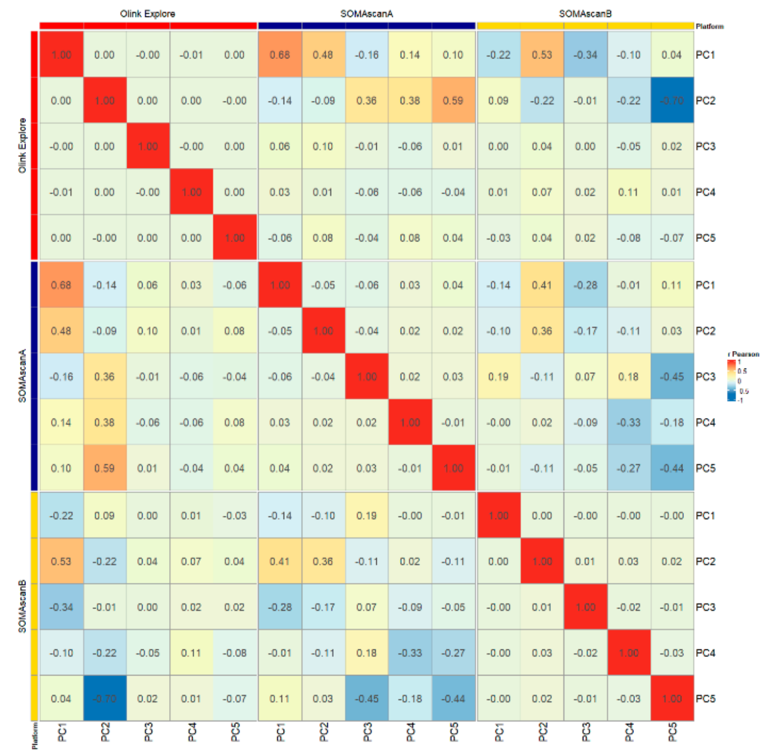

Supplementary Figure S1. Pearson correlation between PCs across proteomic analysis in Olink Explore and SOMAscan.

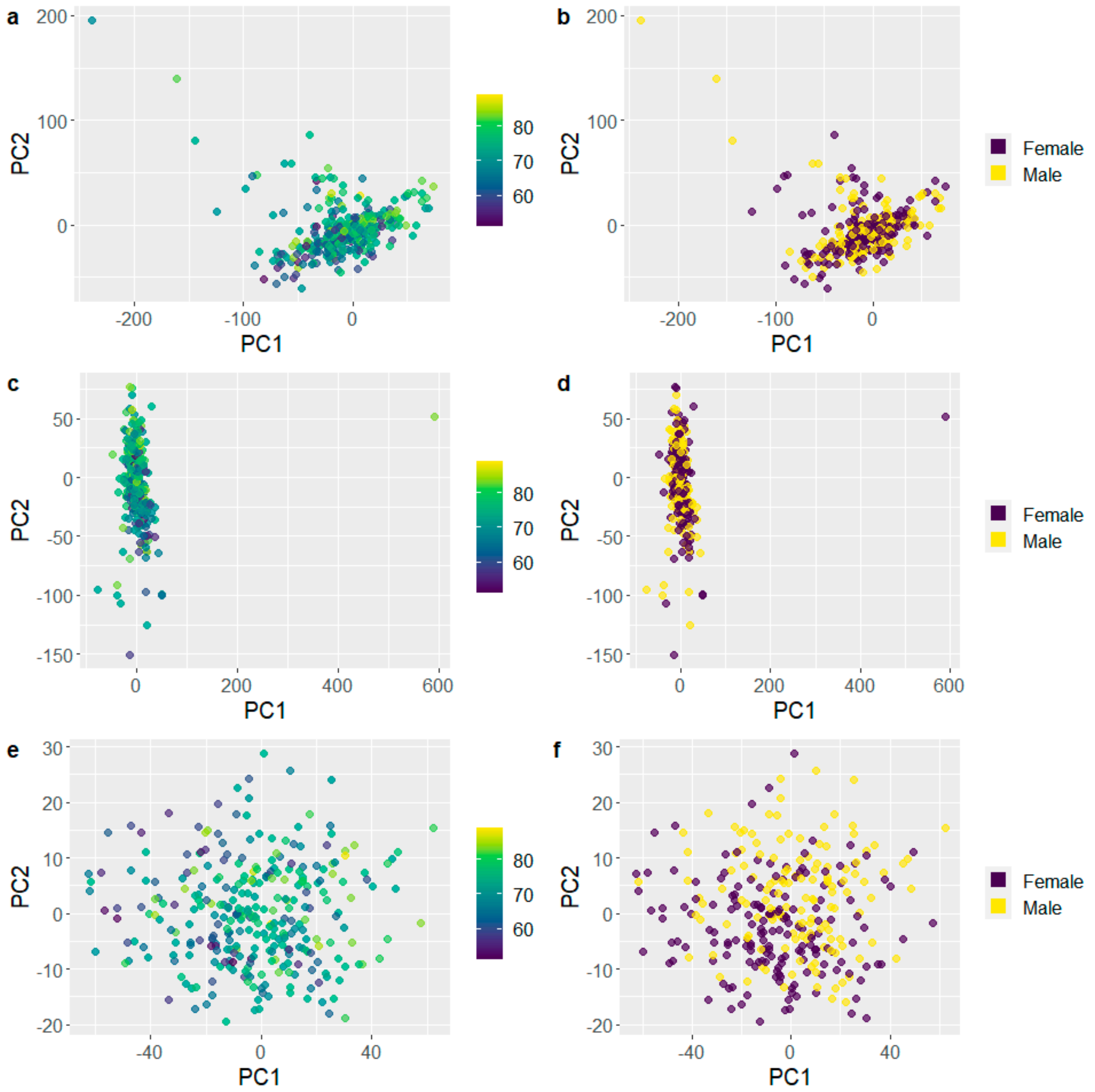

Supplementary Figure S2. Representation of PC1 and PC2 coloured by Age at LP and sex on SOMAscan and Olink datasets. SOMAscanA datasets (a and b), SOMAscanB datasets (c and d) and Olink Explore datasets (e and f). Outlier individuals were not considered in this plot.

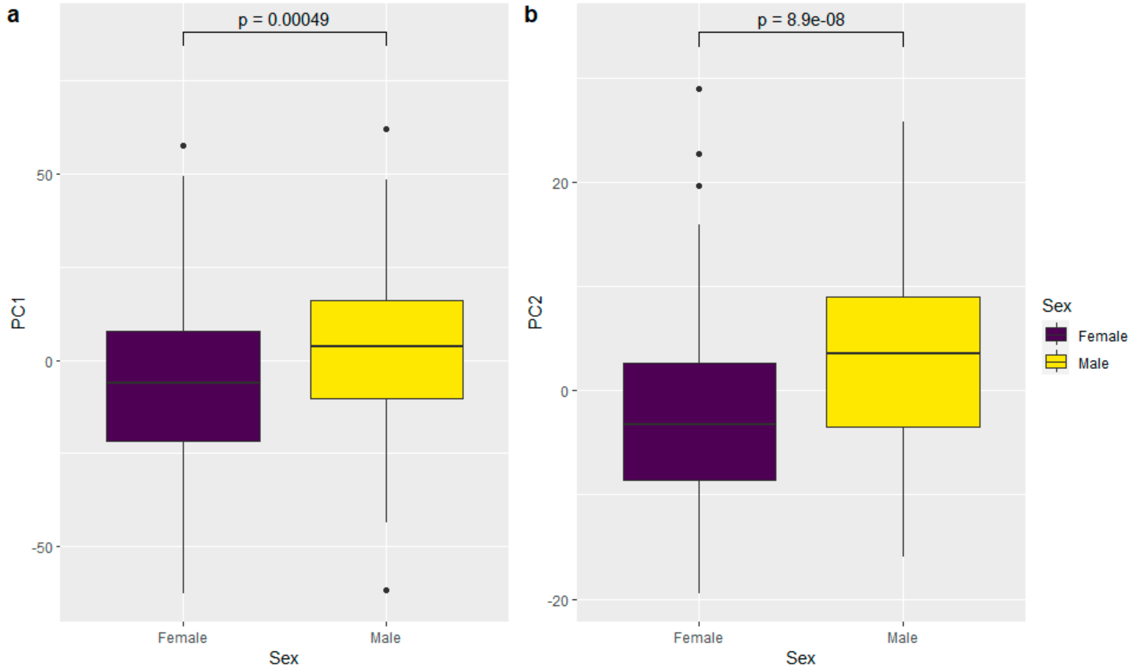

Supplementary Figure S3. Differences in PC levels between sex in the Olink Explore proteomic platform. left: PC1. right: PC2.

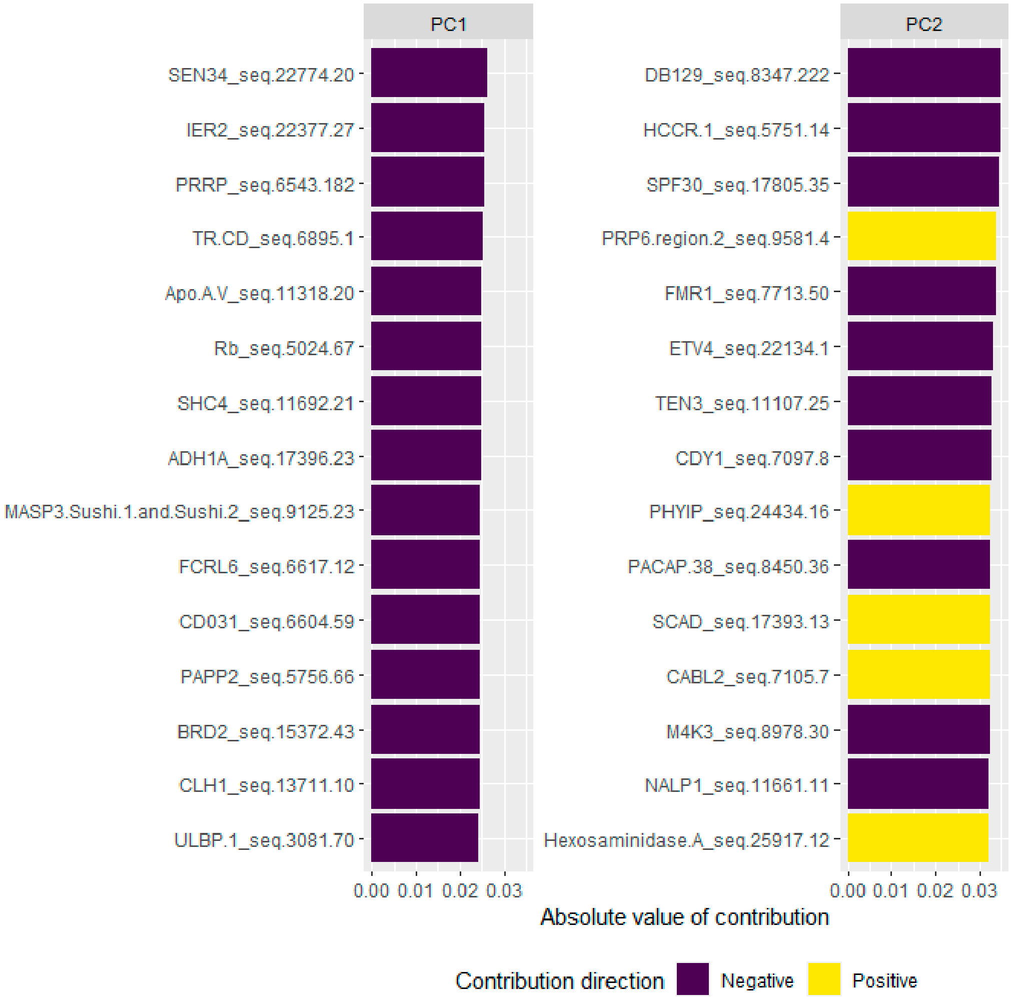

Supplementary Figure S4. Top 15 proteins contributing to PC1 and PC2 in the SOMAscanA dataset.

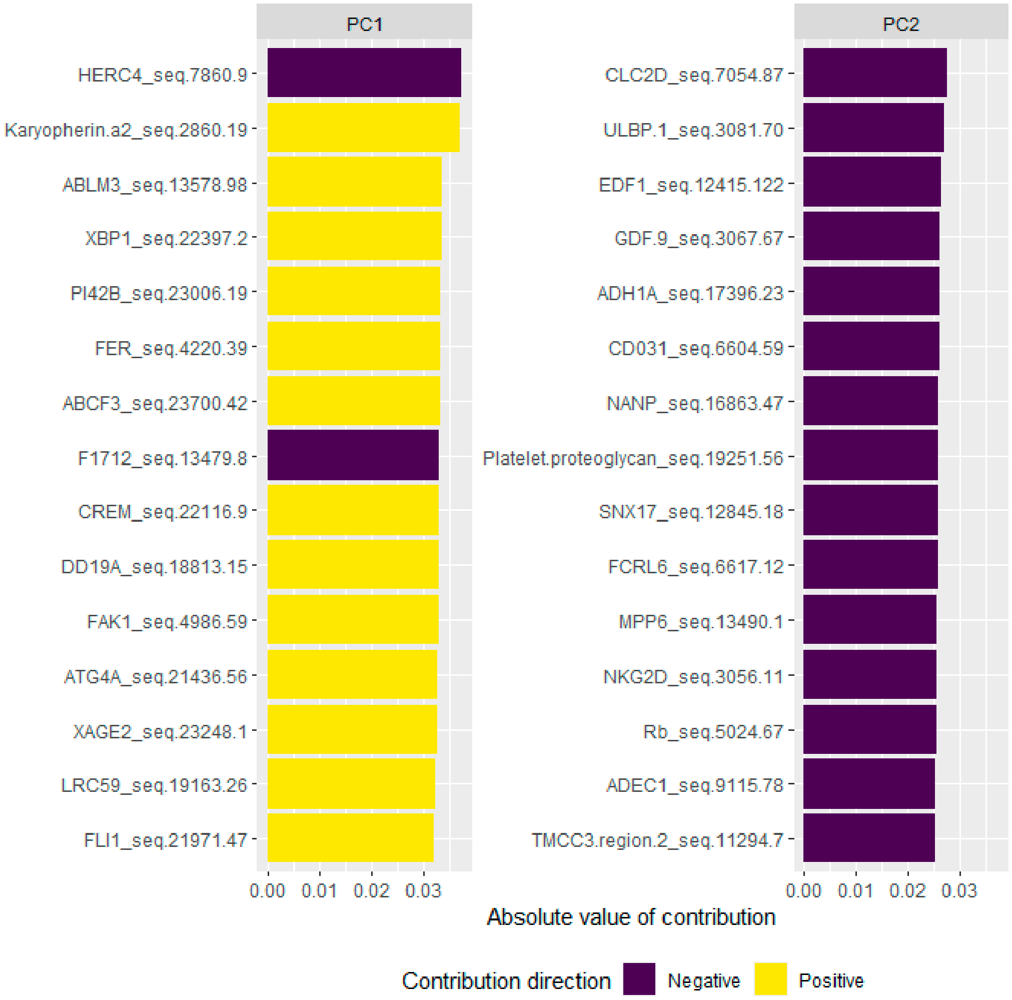

Supplementary Figure S5. Top 15 proteins contributing to PC1 and PC2 in the SOMAscanB dataset.

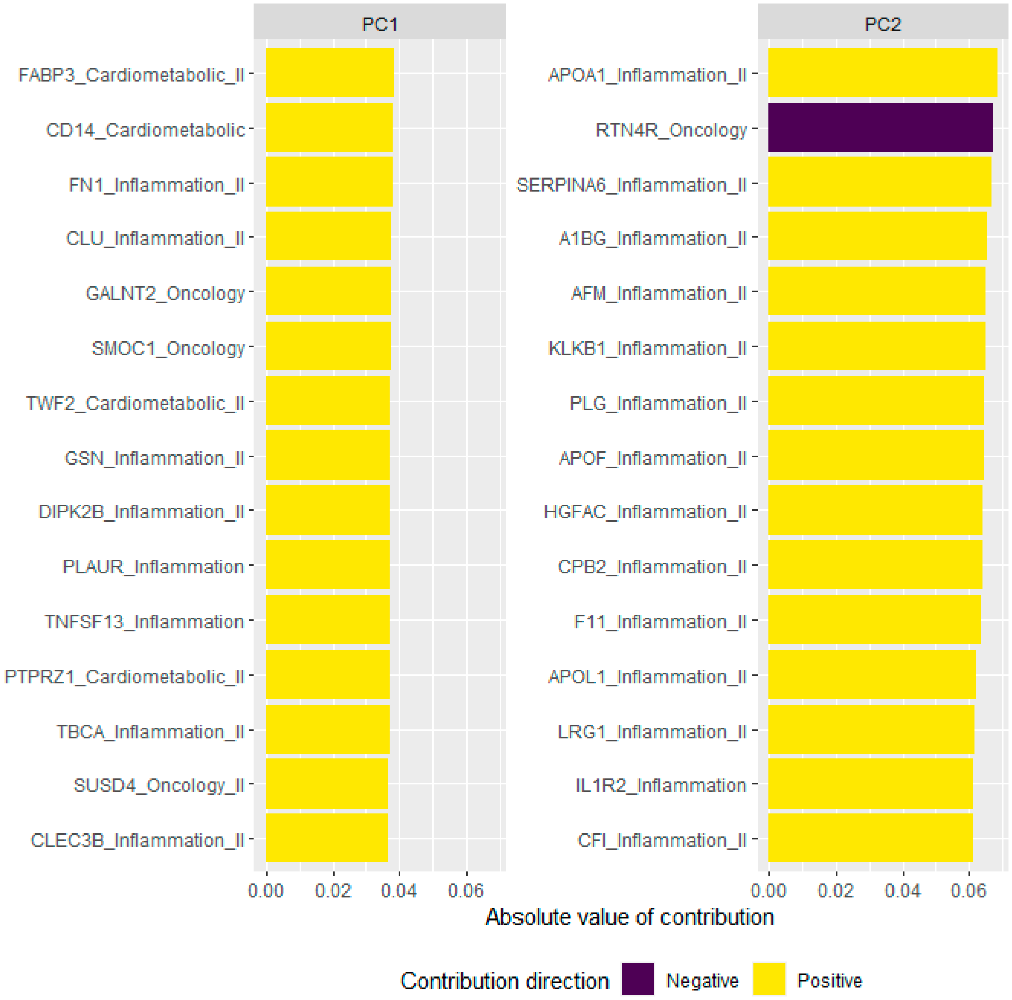

Supplementary Figure S6. Top 15 proteins contributing to PC1 and PC2 in the Olink Explore dataset.

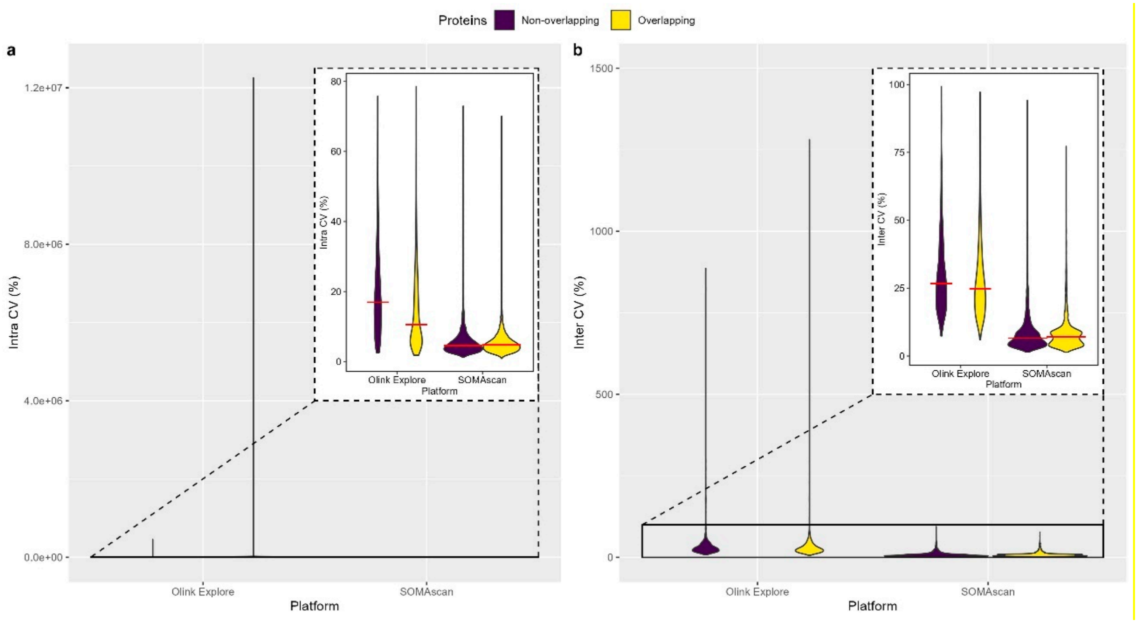

Supplementary Figure S7. Coefficient of variation for SOMAscan and Olink Explore Platforms. a) Intra- and b) Inter-assay coefficient of variation coloured by the overlapping between SOMAscan and Olink assays. The red line in the zoom plot represents the median CV for each platform.

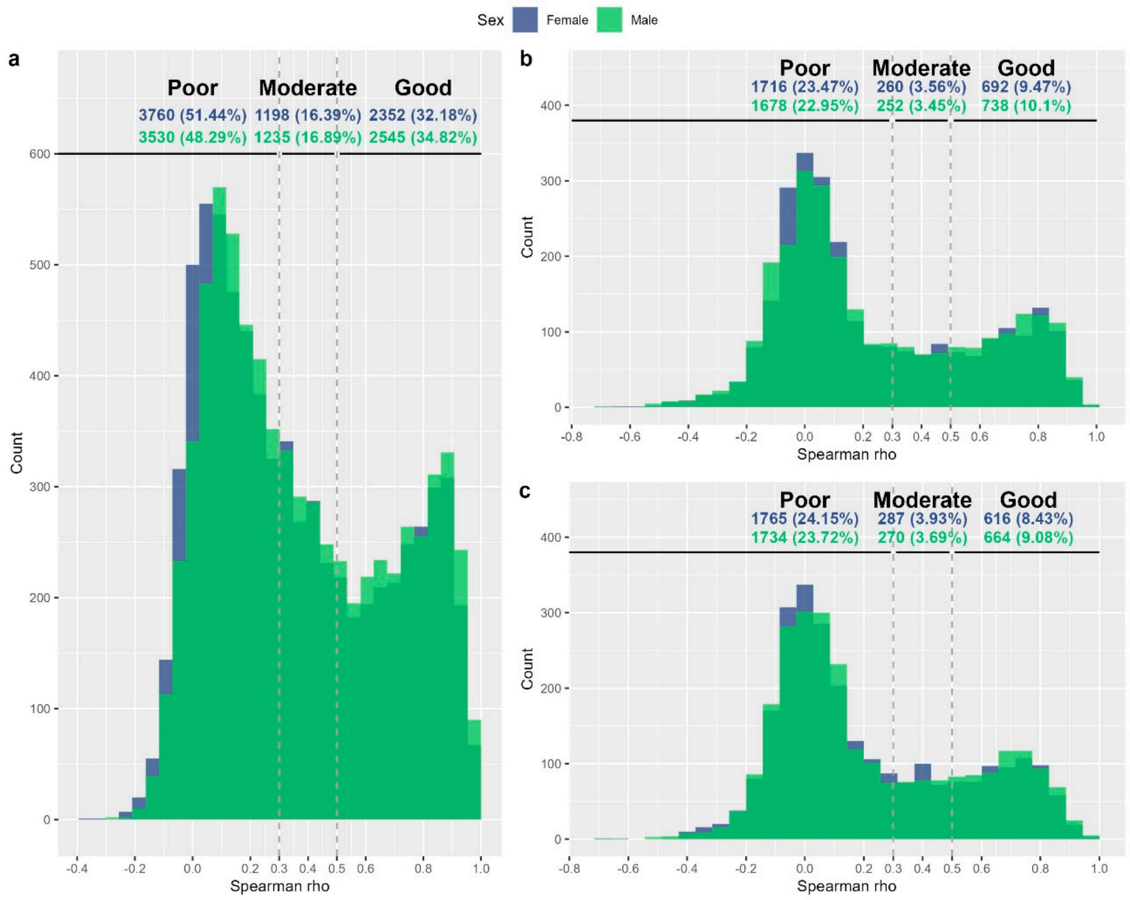

Supplementary Figure S8. Distribution of Spearman's rho values in the correlation analysis stratified by sex, including the sample size of each category [n (%)]. A) Intra-platform correlation between SomaScanA and SomaScanB assays, B) Inter-platform correlation between SomaScanA and Olink Explore, and C) Inter-platform correlation between SomaScanB and Olink Explore platforms. We established three categories: good (rho > 0.5), moderate (0.5 > rho ≥ 0.3) and poor (rho < 0.3).

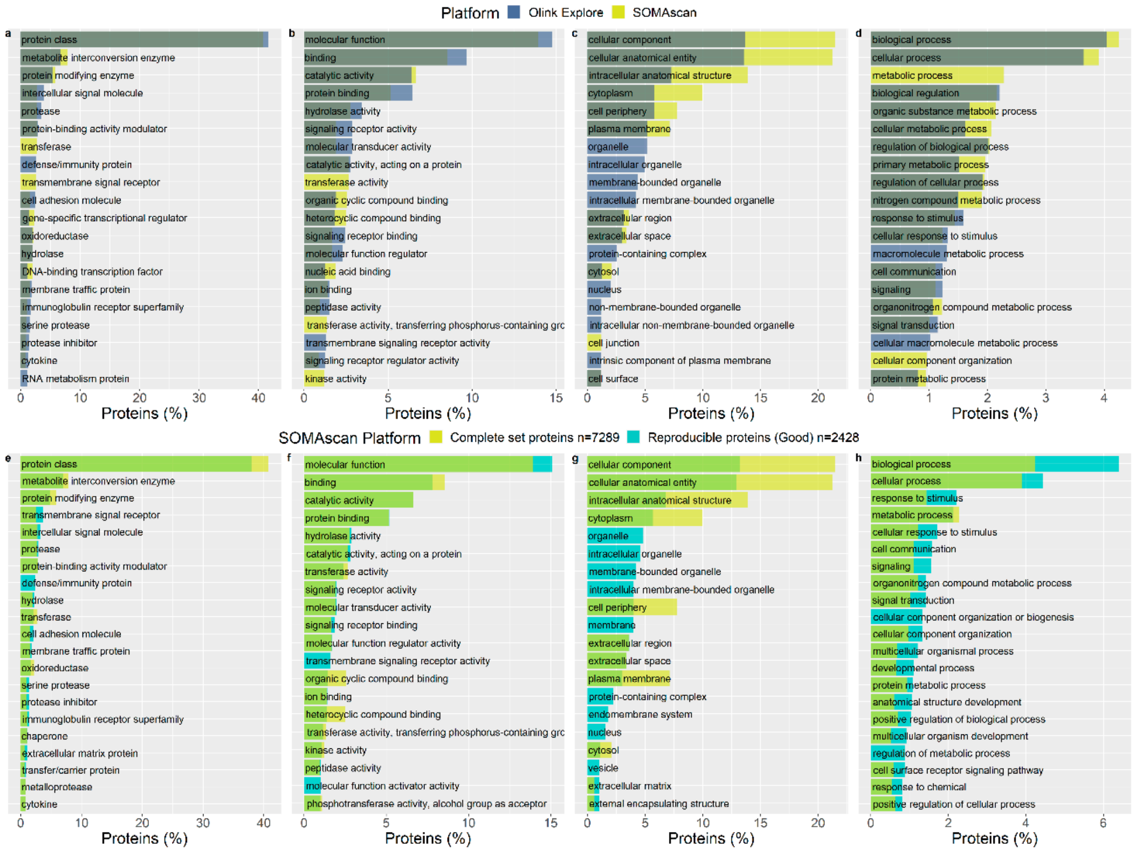

Supplementary Figure S9. **Top 20 significant classifications in PANTHER database (FDR < 0.05).** A) protein class, B) molecular function, C) cellular compartment and D) biological process in Olink® Explore (dark blue; n=2,872) and SOMAscan® (yellow; n=6,218). E) protein class, F) molecular function, G) cellular compartment and H) biological process in the complete set of SOMAscan® proteins (yellow; n=6,218) and reproducible SOMAscan® proteins with Good intra-assay correlation (rho ≥ 0.5) (light blue; n=2,120).

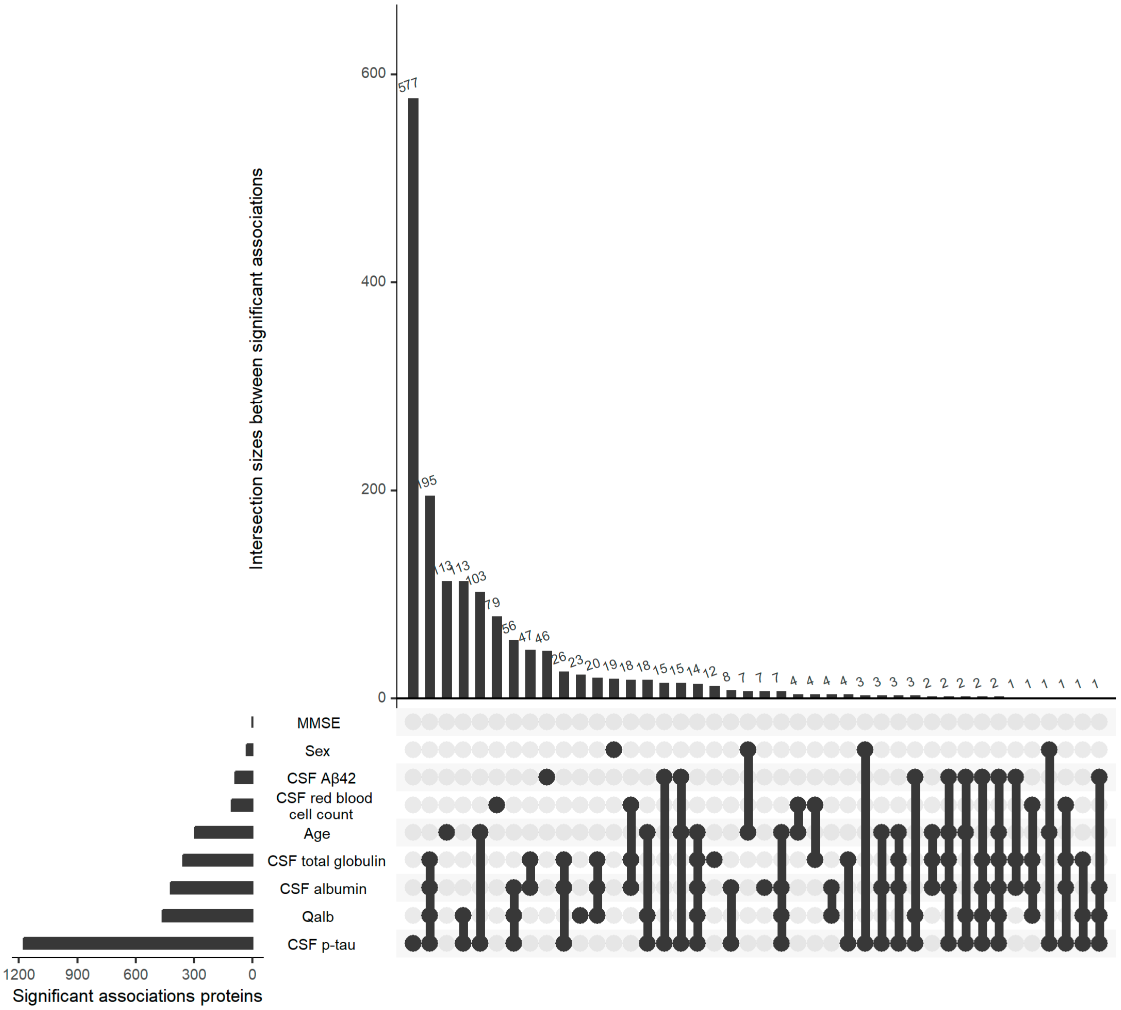

Supplementary Figure S10. Representation of the overlapping significant proteins among the association analyses with CSF Biological Traits, Sample Demographics, and Alzheimer's Disease Endophenotypes. This graph was conducted using the UpSetR R package.

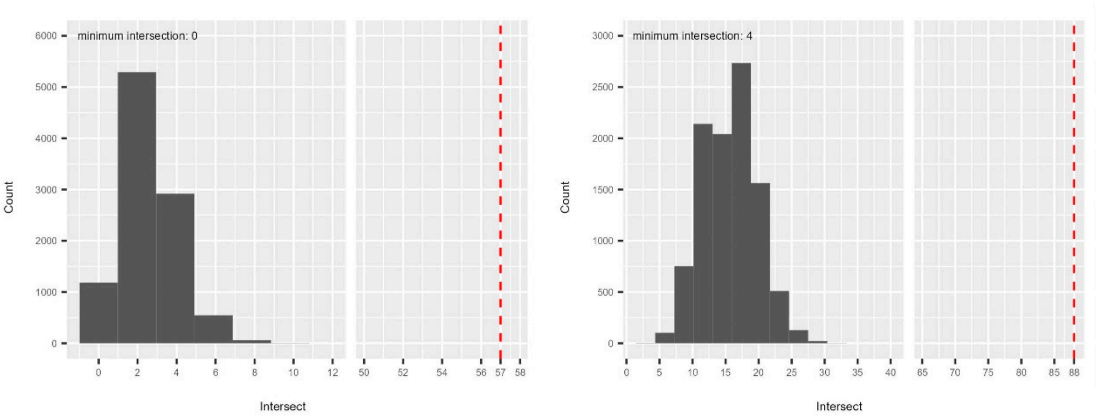

Supplementary Figure S1. Simulation of the overlapping between three sets of proteins. Left) Considering the complete set of SOMAscan proteins n=7,289. Right) Considering only reliable and reproducible proteins with Good intra-assay correlation n=2,428. The x-axis is truncated to provide a better visualization of results. The red dashed line represents the number of overlapping proteins that we found in our analysis.

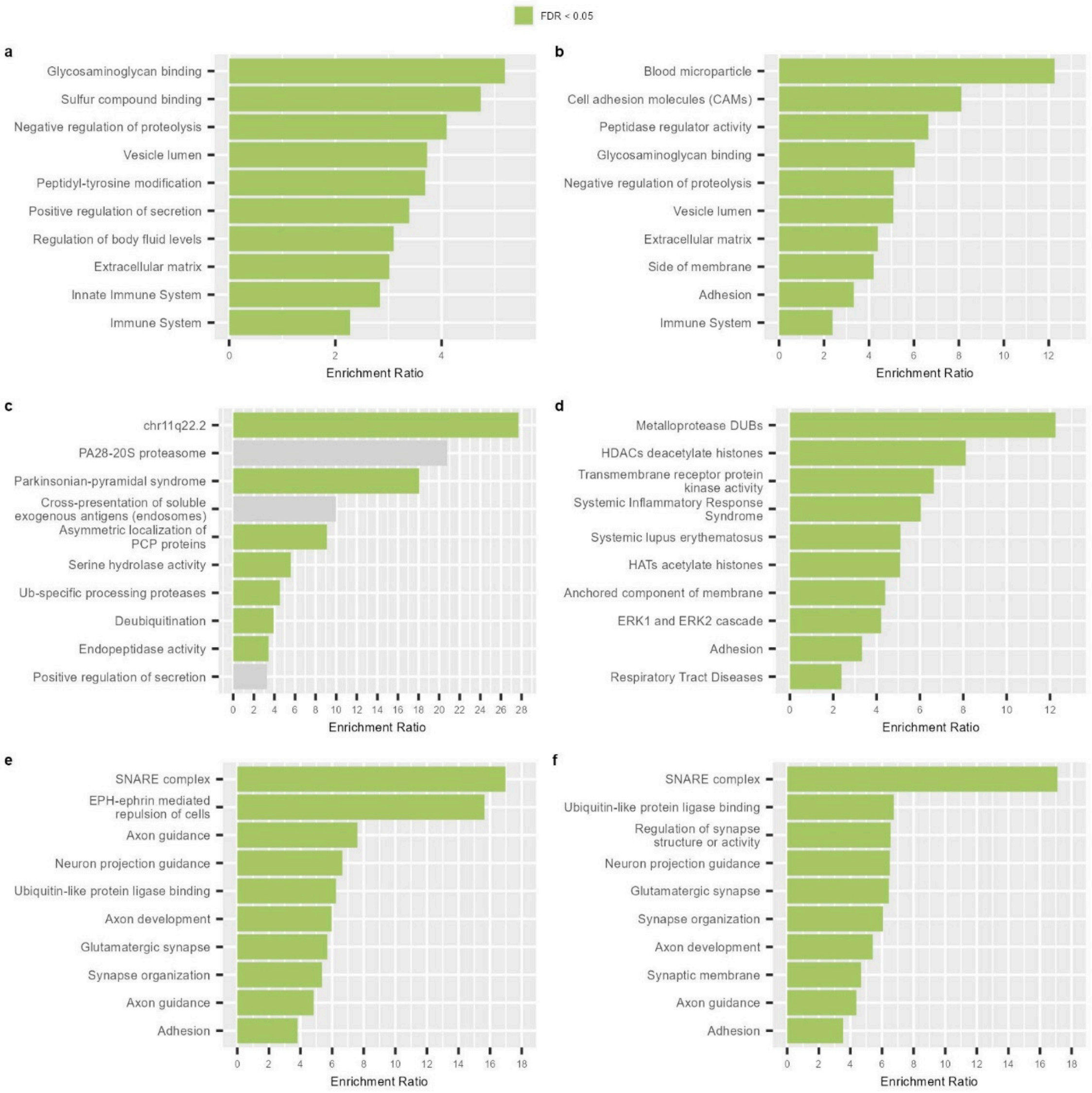

Supplementary Figure S2. Top 10 enrichment analysis of non-intersecting proteins in the top 500 ranking of significant associations of MMSE, CSF Aβ42 and CSF p-tau. A) 332 non-intersecting proteins in the MMSE top ranking considering the complete set of proteins, B) 264 non-intersecting proteins in the MMSE top ranking considering reproducible SOMAscan proteins**,** C) 240 non-intersecting proteins in the CSF Aβ42 top ranking considering the complete set of proteins and D) 190 non-intersecting proteins in the CSF Aβ42 top ranking considering reproducible SOMAscan proteins, E) 315 non-intersecting proteins in the CSF p-tau top ranking considering the complete set of protein, and F) 260 non-intersecting proteins in the CSF p-tau top ranking considering reproducible SOMAscan proteins. We used the WebGestalt tool for the enrichment analysis.

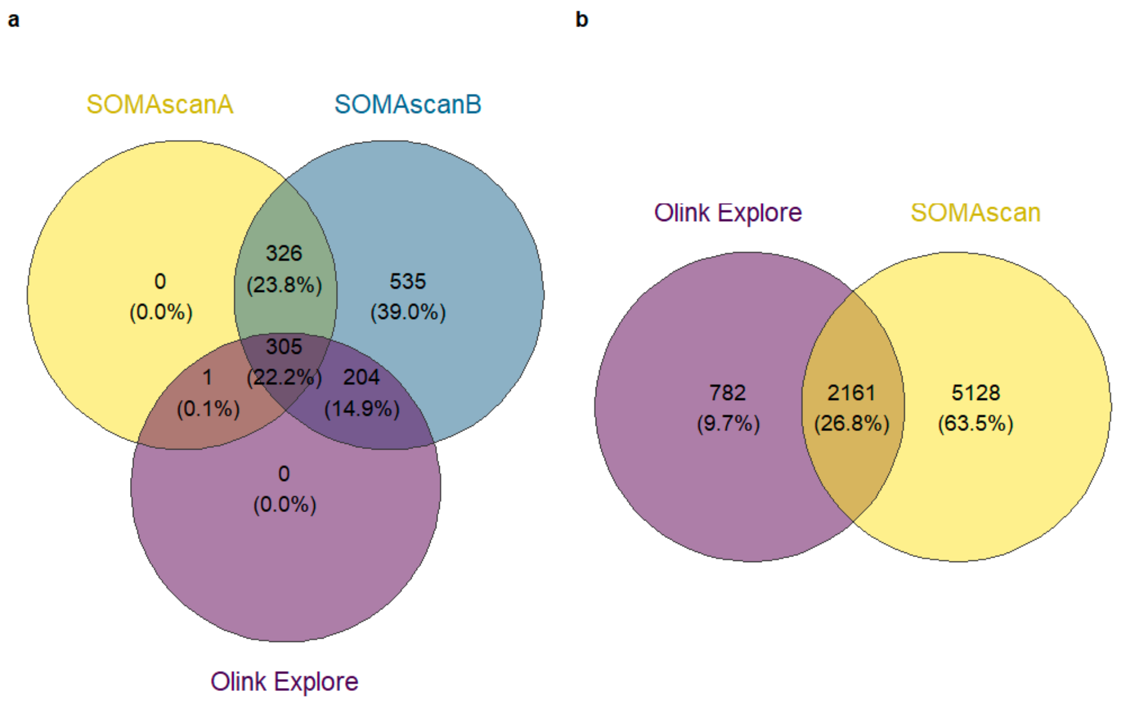

Supplementary Figure S13. Venn diagrams. a) Individuals overlapping in SOMAscanA, SOMAscanB and Olink Explore proteomic analyses. b) Proteins identified in SOMAscan and Olink Explore platforms, we performed the pairing of both platforms by UniProtIDs.
